# Real-world use of finerenone in diabetic kidney disease: eGFR slope analysis with exploratory data on prior MRA use

**DOI:** 10.1007/s13340-025-00839-5

**Published:** 2025-07-25

**Authors:** Yuka Yamao, Miwa Ota, Keizo Kanasaki

**Affiliations:** 1 Faculty of Medicine, Internal Medicine I, Shimane University, 89-1 Enya-Cho, Izumo, Shimane 693-8501 Japan; 2 The Center for Integrated Kidney Research and Advance, Faculty of Medicine, Shimane University, 89-1 Enya-Cho, Izumo, 693-8501 Japan

**Keywords:** Finerenone, Diabetic kidney disease, eGFR slope, Mineralocorticoid receptor antagonist, Real-world evidence, SGLT2 inhibitor

## Abstract

In the post-SGLT2 inhibitor era, residual renal risk remains a major concern in diabetic kidney disease (DKD). Finerenone, a non-steroidal mineralocorticoid receptor antagonist (MRA), has shown cardiovascular and renal benefits in clinical trials. However, its effectiveness in patients previously treated with other MRAs remains unclear. We retrospectively analyzed 73 patients with DKD who were treated with finerenone at Shimane University Hospital between June 2022 and March 2024. The primary outcome was the change in estimated glomerular filtration rate (eGFR) slope before and after finerenone initiation. The mean eGFR slope significantly improved following finerenone initiation (−7.3 to −0.5 mL/min/1.73 m^2^/year, *P* = 0.010). In a sub-analysis of 65 patients with ≥6 months of follow-up, compared to the 1-year pre-treatment period, the decline in eGFR was significantly attenuated (−5.2 to −0.03 mL/min/1.73 m^2^/year, *P* = 0.036), accompanied by a significant reduction in the urinary albumin-to-creatinine ratio (UACR). Notably, one-third of patients had switched from other MRAs, most commonly esaxerenone. In this subgroup, a trend toward eGFR preservation was observed, although the reduction in UACR was not observed. In contrast, MRA-naïve patients exhibited significant improvements in both eGFR slope and UACR. No serious cases of hyperkalemic crisis were observed. These findings highlight the favorable renal effects of finerenone, even in high-risk patients previously managed with other MRAs. Finerenone may offer incremental benefit for eGFR preservation and supports its role in comprehensive DKD management.

## Introduction

Diabetic kidney disease (DKD) remains a leading cause of chronic kidney disease (CKD) that progresses to end-stage kidney disease (ESKD) [1, 2]. In the late twentieth century, renin-angiotensin system (RAS) inhibitors were introduced as a treatment for DKD; however, despite this advance, residual risks have remained a major challenge. Since the approval of captopril, an ACE inhibitor, for the treatment of DKD [3], nearly three decades passed without a significant therapeutic breakthrough. This paradigm changed in 2015 with the EMPA-REG OUTCOME trial [4, 5], which proposed a new hypothesis positioning sodium-glucose cotransporter 2 (SGLT2) inhibitors as effective agents in DKD management [4]. This hypothesis was subsequently validated by the CREDENCE [6], DAPA-CKD [7], and EMPA-KIDNEY [8] trials. These landmark studies marked a major shift in the treatment landscape, ushering in what is now considered the “post–SGLT2 inhibitor era”. Despite the clear renal and cardiovascular benefits of SGLT2 inhibitors, considerable residual risk persists.

Finerenone, a non-steroidal mineralocorticoid receptor antagonist (MRA), has emerged as a promising new option. Two pivotal trials—FIGARO-DKD [9] and FIDERIO-DKD [10]—demonstrated its efficacy in reducing both renal and cardiovascular events in patients with DKD. In comparable patient populations, finerenone appears to offer similar renoprotective and cardioprotective effects to those of the SGLT2 inhibitor canagliflozin, particularly in patients with a urine albumin-to-creatinine ratio (UACR) >300 mg/g [11]. Several studies have also suggested that MRAs reduce albuminuria in DKD. For instance, in a Japanese study, spironolactone reduced UACR by 33% after 8 weeks of treatment in diabetic patients with a UACR between 100 and 2000 mg/g, independent of changes in blood pressure or estimated glomerular filtration rate (eGFR) [12]. Esaxerenone, another non-steroidal MRA, has been shown to lower both UACR and blood pressure in patients with diabetes-associated CKD [13]. However, unlike finerenone, neither spironolactone nor esaxerenone has demonstrated definitive benefits on hard renal outcomes in large-scale trials. For example, the BARACK-D trial evaluating spironolactone (25 mg) in CKD patients (*n* = 1372; mean eGFR 43.5 ml/min/1.73 m^2^; 24% with diabetes; median UACR 13 mg/g) showed no clinical benefit and an increased risk of adverse events [14].

While all MRAs act on the same molecular target—the mineralocorticoid receptor (MR)—they differ in pharmacodynamic and pharmacokinetic properties. Spironolactone, a steroidal MRA, also binds androgen and progesterone receptors and is metabolized into multiple active compounds that are excreted renally [15, 16], a limitation in patients with impaired kidney function due to potential metabolite accumulation. Esaxerenone, though non-steroidal and promising in small studies, is primarily an antihypertensive agent and differs mechanistically from finerenone, which exerts minimal effects on blood pressure [15]. In preclinical studies, finerenone demonstrated superior protective effects on podocytes and proximal tubules compared to spironolactone and amiloride in DOCA-salt hypertensive rats [17].

Here, we report our clinical experience indicating that finerenone confers renal protection in patients with DKD, with a potentially more favorable effect on eGFR slope compared to other MRAs.

## Method

### Patients

We retrospectively analyzed 73 patients who were administered finerenone at the Department of Endocrinology and Metabolism, Shimane University Hospital, between June 2022 and March 2024, and for whom eGFR data were available for at least 3 months after initiation. This study was conducted with the approval of the Institutional Review Board of the Faculty of Medicine, Shimane University (Approval No. 20230328-1, approved at 2023.5.8).

### Data collection

Clinical data were extracted retrospectively from electronic medical records. The eGFR slope (ΔeGFR: annual change in eGFR (ml/min/1.73m^2^/year)) was calculated by the slope of a linear approximation model. The collected data will be stored on a computer within the Department of Endocrinology and Metabolism at Shimane University Hospital, which is not accessible from external sources. Security measures will be implemented on the computer, and access will be restricted to authorized researchers via password protection. A subject identification list will be stored separately from the data in a locked location under the responsibility of the principal investigator. No data will be taken outside the facility. All research-related data and associated documents (including procedural records) will be retained for 10 years following the final publication of the study. Thereafter, when retention is no longer necessary, the materials will be securely discarded (deleted) in a manner that ensures individuals cannot be identified.

### Statistical analysis

Changes in clinical and laboratory parameters before and after finerenone administration was compared using paired *t*-tests. We examined differences in clinical and laboratory parameters among these groups using Pearson’s chi-square test and the McNemar test. Patients were grouped according to whether they switched from another MRA. Changes in clinical and laboratory parameters before and after finerenone administration were compared using paired *t*-tests. A two-sided significance level of 5% was used for all statistical analyses. UACR values were log-transformed prior to analysis. All statistical analyses were conducted using SPSS version 29.

## Results

### Patients characteristics

We retrospectively analyzed patients who were prescribed finerenone between June 2022 and March 2024 at Shimane University Hospital and who had at least 3 months of follow-up data available. Patients characteristics were shown in Table 1. A total of 73 patients with DKD were included (32.9% female), with a mean age of 64.0 ± 12.6 years and a mean BMI of 25.6 ± 4.9 kg/m^2^. The mean systolic and diastolic blood pressures were 134 ± 21 mmHg and 76 ± 13 mmHg, respectively. According to the Japanese Classification of Diabetic Nephropathy, the distribution of disease stages was as follows: Stage 1 (normoalbuminuria), 5.5%; Stage 2 (microalbuminuria), 21.9%; Stage 3 (macroalbuminuria), 46.6%; and Stage 4 (eGFR <30 ml/min/1.73 m^2^), 23.3%. All patients were treated with an angiotensin converting enzyme (ACE) inhibitor or angiotensin receptor blocker (ARB) unless contraindicated. At the time of initiating finerenone, 90.4% of patients were already on SGLT2 inhibitors, and more than half were receiving GLP-1 receptor agonists. Additionally, 28.8% (21 cases) of patients had previously been treated with other MRAs (esaxerenone 18 cases, spironolactone 2 cases, and eplerenone 1 case). Serum potassium levels were well controlled (4.4 ± 0.4 mmol/L), and the mean creatinine-based eGFR was 48.0 ± 24.8 ml/min/1.73 m^2^ (Table 1). The eGFR slope prior to finerenone prescription, calculated using all available data in the electronic health records (ΔeGFR_prewhole_), was −7.3 ± 20.8 ml/min/1.73 m^2^/year. When restricted to the 1-year period immediately prior to finerenone initiation (ΔeGFR_pre1year_), the slope was −5.5 ± 30.0 ml/min/1.73 m^2^/year. Urinary protein/creatinine ratio (UPCR) and urinary albumin/creatinine ratio (UACR) were measured in 50 and 57 patients, with mean values of 2.6 ± 4.1 g/gCr and median values 494 (9.6–14,382.9) mg/gCr (Table 1).

**Table 1:** Patients characteristics

Age	64.0 ± 12.6
Male (%)	67.1
BMI (kg/m^2^)	25.6 ± 4.9 (*n*=20)
Sys BP(mmHg)	134 ± 21 (*n*=66)
Dia BP(mmHg)	76 ± 13 (*n*=65)
Diabetic nephropathy stage 1/2/3/4 (%)	5.5/21.9/46.6/23.3 (*n*=71)
SGLT2 inhibitor（%）	90.4
GLP-1receptor agonist（%）	57.5
Switched from other MRAs（%）	28.8（Esaxerenone 18, Spironolactone 2, Eplerenone 1）
K^+^ binders （%）	12.3
HbA1c (%)	7.5 ± 1.3 (*n*=73)
Hb (g/dL)	13.6 ± 2.2 (*n*=67)
TG (mg/dL)	182 ± 123 (*n*=68)
HDL-C (mg/dL)	55 ± 16 (*n*=67)
LDL-C (mg/dL)	93 ± 26 (*n*=66)
UA (mg/dL)	5.8 ± 1.7 (*n*=61)
K (mmol/L)	4.4 ± 0.4 (*n*=71)
eGFR (ml/min/1.73m^2^)	48.0 ± 24.8 (*n*=73)
ΔeGFR_prewhole_	−7.3 ± 20.8 (*n*=73)
ΔeGFR_pre1year_ (ml/min/1.73m^2^/year)	−5.5 ± 30.0 (*n*=73)
UPCR (g/gCr)	2.6 ± 4.1 (*n*=50)
UACR (mg/gCr)^a^	494.9（9.6–14382.9）(*n*=57)

Mean ± S.D.

^a^Median(min–max)

*BMI* Body mass index, *Sys BP* and *Dia BP* indicated systolic and diastolic blood pressure, *SGLT2* Sodium-glucose cotransporter 2, *Hb* Hemoglobin, *TG* Triglyceride, *HDL* High-density lipoprotein, *LDL* Low density lipoprotein, *K* Potassium, *UA* Uric acid, *eGFR* Estimated glomerular filtration rate, *UPCR* Urine protein to creatinine ratio, *UACR* Urine alubmin to creatinine ratio

When all data were included, finerenone initiation was significantly associated with attenuation in the decline of eGFR (ΔeGFR_prewhole_: −7.3 ± 20.8 vs. ΔeGFR_postwhole_: −0.5 ± 12.3 ml/min/1.73 m^2^/year, *P* = 0.010) (Table 2). It should be noted that ΔeGFR_prewhole_ may overestimate the effect of finerenone, as many patients had already initiated SGLT2 inhibitors or other therapies prior to finerenone. Therefore, to better evaluate the impact of finerenone within this constraint, we analyzed ΔeGFR using the closest available data point within 1 year prior to treatment initiation (ΔeGFR_pre1yeear_: −5.5 ± 30.0 ml/min/1.73 m^2^/year) instead of ΔeGFR_prewhole_, similar trend of eGFR preservation was found. These results suggest that current multidisciplinary, evidence-based interventions may help slow DKD progression. While UPCR remained unchanged (*n* = 46), UACR significantly decreased following finerenone initiation (*n* = 45, 436.4 vs. 258.2 mg/gCr, *P* = 0.002). Systolic blood pressure was trended suppressed (*n* = 59, 133.7 ± 20.0 vs.129.4 ± 18.3, *P* = 0.053) and BMI was significantly suppressed (*n* = 65, 25.8 ± 4.8 vs. 25.3 ± 4.9, *P* = 0.006). Percent prescription of potassium binders was not altered (12.3% vs. 13.7%).

**Table 2:** eGFR slope in all patients with whole periods

*n*=73	Pre	Post	*P* value
Periods (months)	98.9 ± 56.5	8.8 ± 4.6	
ΔeGFR (ml/min/1.73m^2^/year)	−7.3 ± 20.8	−0.5 ± 12.3	0.010

Mean ± S.D.

Furthermore, post-finerenone follow-up duration remains limited, given the drug market availability since June 2022. Therefore, we compared ΔeGFR_pre1year_ to ΔeGFR over a minimum of 6 months post-treatment (ΔeGFR_post6M~_) (Table 3). Among 65 cases, significant eGFR preservation was observed (ΔeGFR_pre1year_: −5.2 ± 12.6 vs. ΔeGFR_post6M~_: −0.03 ± 10.7 ml/min/1.73 m^2^/year, *P* = 0.036), accompanied by a significant reduction in UACR (*n* = 42, 428.9 vs. 261.4 mg/gCr, *P* = 0.007). Blood pressure was trended suppressed and BMI was significantly suppressed. Percent prescription of potassium binders was not altered (Table 3).

**Table 3:** Changes in clinical parameters in patients (Analysis limited to individuals with post-treatment data available for at least 6 months. Pre-treatment data represent the closest available value within 1 year prior to treatment

*n*=65	Pre	Post	*P* value
Periods (months)	10.7 ± 2.6	9.4 ± 4.6	
ΔeGFR (ml/min/1.73m^2^/year)	−5.2 ± 12.6	−0.03 ± 10.7	0.036
UPCR (g/gCr)	2.3 ± 3.5	2.0 ± 4.4	0.457(*n* = 43)
UACR (mg/gCr)^a^	428.9（9.6–14382.9）	261.4（9.5–18982.8）	0.007(*n* = 42)
K (mmol/L)	4.4 ± 0.4	4.5 ± 0.4	0.106 (*n* = 62)
HbA1c (％)	7.5 ± 1.4	7.2 ± 1.3	0.085 (*n* = 64)
Sys BP (mmHg)	132.1 ± 18.7	128.3 ± 17.5	0.109 (*n* = 53)
Dia BP (mmHg)	75.6 ± 12.3	72.9 ± 11.3	0.109(*n* = 51)
BMI	26.0 ± 4.8	25.5 ± 5.0	0.005 (*n* = 59)
K^+^ binders (%)	12.3	15.3	0.625

Mean ± S.D.

^a^Median(min–max)

*BMI* Body mass index, *Sys BP* and *Dia BP* indicated systolic and diastolic blood pressure, *SGLT2* Sodium-glucose cotransporter 2, *Hb* Hemoglobin, *TG* Triglyceride, *HDL* High-Density Lipoprotein, *LDL* Low density lipoprotein, *K* Potassium, UA Uric acid, eGFR estimated glomerular filtration rate, *UPCR* Urine protein to creatinine ratio, *UACR* Urine alubmin to creatinine ratio

Approximately one-third of patients initiating finerenone had switched from another MRA, most commonly from esaxerenone. These patients tended to be younger, with higher BMI, elevated triglycerides, lower HDL-C, and a lower eGFR with higher potassium levels, indicating a high-risk metabolic and renal profile (Table 4). Furthermore, all patients in this subgroup were already on SGLT2 inhibitors (100%), and most of them were also treated with GLP-1 receptor agonists (76.2%), reflecting prior intensive multidisciplinary management. Potassium binders are more common in MRA switch group (23.8% vs 6.8%) (Table 4).

**Table 4:** Patient characteristics with or without a switch from other MRAs to Finerenone, limited to individuals with data available for at least 6 months after initiation

	Switch from other MRA		
	Yes (*n*=21)	No (*n*=44)	*P* value
Age	59.7 ± 13.2	64.5 ± 11.3	0.133
Male(％)	76.2	65.9	0.401
BMI (kg/m^2^)	27.6 ± 3.8	24.9 ± 5.2	0.040
Sys BP (mmHg)	131 ± 20	134 ± 20	0.567 （*n* =21, 37）
Dia BP (mmHg)	77 ± 14	75 ± 13	0.566 （*n* =20, 37）
Diabetic nephropathy stage 1/2/3/4（%）	0/20.0/40.0/40.0	6.8/25.0/54.5/13.6	0.091
SGLT2 inhibitor（%）	100.0	86.3	0.076
GLP-1receptor agonist（%）	76.2	47.7	0.030
Potassium binders （%）	23.8	6.8	0.051
HbA1c (％)	7.2 ± 1.2	7.6 ± 1.4	0.323
Hb (g/dL)	13.3 ± 2.1	14.0 ± 2.1	0.245（*n* =20, 39）
TG (mg/dL)	259 ± 163	156 ± 90	0.014（*n* =20, 41）
HDL-C (mg/dL)	47 ± 11	56 ± 15	0.037（*n* =19, 40）
LDL-C (mg/dL)	96 ± 24	90 ± 24	0.386（*n* =19, 40）
K (mmol/L)	4.6 ± 0.5	4.3 ± 0.4	0.020（*n* =20, 43）
eGFR (ml/min/1.73m^2^)	39.9 ± 24.0	52.7 ± 24.0	0.049
ΔeGFR_pre1year_ (ml/min/1.73m^2^/year)	−2.3 ± 6.2	−6.5 ± 14.5	0.210
UPCR (g/gCr)	2.8 ± 3.9	1.8 ± 3.1	0.324（*n* =17, 29）
UACR (mg/gCr)^a^	965.1（70.5–4646.5）	526.4（9.6–14382.9）	0.553（*n* =15, 38）

Mean ± S.D.

^a^Median(min–max)

*BMI* Body mass index, *Sys BP* and *Dia BP* indicated systolic and diastolic blood pressure, *SGLT2* Sodium-glucose cotransporter 2, *Hb* Hemoglobin, *TG* Triglyceride, *HDL* High-density lipoprotein, *LDL* Low density lipoprotein, *K* Potassium, *UA* Uric acid, *eGFR* estimated glomerular filtration rate, *UPCR* Urine protein to creatinine ratio, *UACR* Urine alubmin to creatinine ratio

**Fig. 1:**
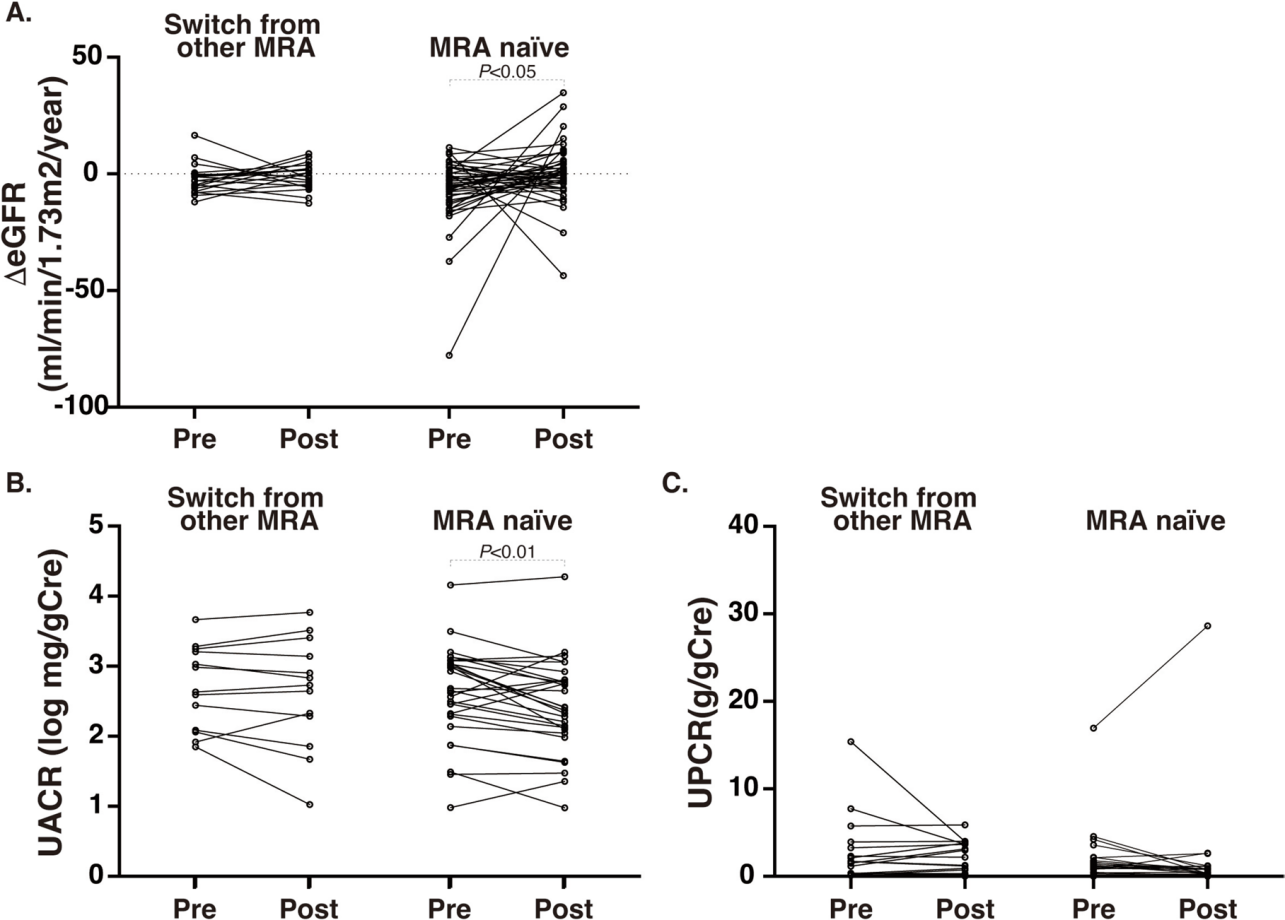
The influence of Finerenone on renal parameters with or without prior MRAs. **A** ΔeGFR alteration in patients with or without prior MRA use. Alteration UACR **(B)** or UPCR **(C)**. *UPCR* Urine protein to creatinine ratio, *UACR* Urine albumin to creatinine ratio. Pre: 1-year period immediately prior to finerenone initiation, Post: at least 6 months after Finerenone initiation

In subgroup analysis comparing patients with and without prior MRA use, among patients who were followed for more than 6 months, those without prior MRA use showed a significant eGFR preservation (ΔeGFR_pre1year_: −6.5 ± 14.5 vs. ΔeGFR_post6M~_: −0.4 ± 12.4 ml/min/1.73 m^2^/year, *P* = 0.049) and a significant reduction in UACR (*n* = 29, 451.4 vs. 258.2 mg/gCr, *P* = 0.009) (Fig. 1A and B). Among those without prior MRA, systolic blood pressure and BMI were also significantly suppressed (Table 5). In contrast, patients previously treated with another MRA exhibited a similar trend in eGFR (ΔeGFR_pre1year_: −2.3 ± 6.2 vs. ΔeGFR_post6M~_: −0.9 ± 5.4 ml/min/1.73 m^2^/year, *P* = 0.444), but no significant change in UACR (*n* = 13, 428.9 vs. 536.9 mg/gCr) (Fig. 1A and B) associated with non-significant alteration in blood pressure and BMI. No significant change in UPCR was observed under either condition (Fig. 1C).

**Table 5:** Changes in parameters before and after the initiation of finerenone treatment, with or without switching from other MRAs (Analysis limited to individuals with post-treatment data available for at least 6 months. Pre-treatment data represent the closest available value within 1 year prior to treatment initiation.)

	Switch from other MRA					
	Yes (*n*=21)			No (*n*=44)		
	Pre	Post	*P* value	Pre	Post	*P* value
Periods (months)	10.6 ± 3.1	11.3 ± 6.4		10.7 ± 2.4	8.5 ± 3.1	
ΔeGFR	−2.3 ± 6.2	−0.9 ± 5.4	0.444	−6.5 ± 14.5	−0.4 ± 12.4	0.049
UPCR (g/gCr)	2.8 ± 3.9	2.2 ± 1.8	0.450 (*n*=17)	1.9 ± 3.3	1.8 ± 5.5	0.768 (*n*=26)
UACR (mg/gCr)^a^	428.9 (70.5–4646.5)	536.9 (10.6–5866.2)	0.444 (*n*=13)	451.4	258.2	0.009 (*n*=29)
K (mmol/L)	4.6 ± 0.5	4.7 ± 0.4	0.720	4.3 ± 0.3	4.5 ± 0.4	0.074
HbA1c (％)	7.2 ± 1.2	6.8 ± 1.1	0.064	7.6 ± 1.4	7.4 ± 1.4	0.367
Sys BP (mmHg)	132.3 ± 20.2	136.0 ± 16.6	0.325 (*n*=18)	132.0 ± 18.1	124.3 ± 16.8	0.010 (*n*=35)
Dia BP (mmHg)	77.1 ± 14.3	73.9 ± 10.3	0.174 (*n*=18)	74.7 ± 11.2	72.3 ± 11.9	0.292 (*n*=33)
BMI	27.3 ± 3.6	26.7 ± 3.7	0.099	25.3 ± 5.3	24.8 ± 5.4	0.022 (*n*=39)
K^+^ binders (%)	23.8	33.3	0.500	6.8	6.8	1.000

Mean ± S.D.

^a^Median(min–max)

*BMI* Body mass index, *Sys BP* and *Dia BP* indicated systolic and diastolic blood pressure, *K* Potassium, *eGFR* estimated glomerular filtration rate, *UPCR* Urine protein to creatinine ratio, *UACR* Urine alubmin to creatinine ratio

These findings suggest that finerenone may offer superior renal protection in terms of eGFR slope. However, its effect on albuminuria may be comparable to other MRAs, particularly in patients who have already undergone intensive prior treatment.

## Discussion

In this retrospective study, we evaluated the real-world clinical effectiveness of finerenone in patients with DKD. Our findings suggest that the addition of finerenone to comprehensive, evidence-based management significantly ameliorated the decline in eGFR. Interestingly, even in patients switched from other MRAs, finerenone appeared to preserve renal function based on eGFR slope analysis, despite no significant change in UACR.

The concept of MR antagonism as a therapeutic strategy for organ protection, particularly in cardiovascular disease, has been established for decades. The Randomized Aldactone Evaluation Study (RALES) demonstrated that spironolactone, beyond its diuretic effects, reduced all-cause mortality by 30% and significantly lowered cardiovascular mortality and hospitalization rates in patients with severe heart failure (left ventricular ejection fraction ≤ 35%, NYHA class III or IV) [18]. Subsequent studies confirmed the efficacy of second-generation MRA eplerenone in patients with both severe and moderate heart failure [19, 20]. More recently, third-generation MRA finerenone has also demonstrated cardiovascular benefit in trials such as FINEARTS-HF [21], FIGARO-DKD [9], and FIDELIO-DKD [10]. These results collectively support the role of MRAs in cardiovascular protection in patients with established heart disease, heart failure, and advanced DKD.

In contrast, renal outcomes associated with MRAs have historically been inconsistent. A large health insurance database analysis in Taiwan involving over 14,000 patients with CKD stages 3–4 revealed that spironolactone use was associated with a 34% reduction in ESKD incidence, albeit with increased hospitalizations due to hyperkalemia [22]. However, in pre-dialysis patients with stage 5 CKD, spironolactone use correlated with increased all-cause mortality and heart failure-related hospitalization [23]. In DKD populations specifically, while reductions in albuminuria have been consistently observed [12, 13, 24–27], robust evidence on hard renal outcomes was lacking until the publication of the FIGARO-DKD and FIDELIO-DKD trials. Notably, in Japan, eplerenone is contraindicated in patients with albuminuric nephropathy or advanced DKD. Furthermore, sub-analyses of the Japanese populations in the FIDELIO-DKD and FIGARO-DKD studies revealed unclear, and possibly adverse, renal effects [28]. Thus, in light of these uncertainties and limited clinical experience, real-world validation of finerenone’s efficacy in DKD is of significant importance.

Our analysis demonstrated a marked improvement in eGFR decline after finerenone administration (ΔeGFR_prewhole_: −7.3 ± 20.8 vs. ΔeGFR_postwhole_: −0.5 ± 12.3 ml/min/1.73 m^2^/year, *P* = 0.010). A similar trend was observed when comparing ΔeGFR over the year prior to finerenone to that in the post-treatment period of ≥6 months (ΔeGFR_pre1year_ vs. ΔeGFR_post6M~_), with accompanying reductions in UACR. As shown in Table 1, the majority of patients receiving finerenone were already on SGLT2 inhibitors, and over half were treated with GLP-1 receptor agonists. The combination of SGLT2 inhibitor, GLP-1RA, and finerenone has been hypothesized to reduce kidney-related events by up to 58% [29]. While our study is a single-center, observational analysis and not conclusive, it provides strong support for current evidence-based, intensive DKD management strategies incorporating finerenone.

Interestingly, BMI was significantly reduced during the post-finerenone period, particularly in the MRA-naïve group. However, we do not consider this reduction to reflect a direct metabolic effect of finerenone. Rather, we suspect that the observed change may be due to hemodynamic effects, such as improved management of fluid overload. Supporting this notion, the FINEARTS-HF trial clearly demonstrated that finerenone exerted beneficial effects in patients with heart failure and mildly reduced or preserved ejection fraction [21], highlighting its impact on fluid regulation. Alternatively, renal protection by finerenone with UACR suppression, may have contributed to more appropriate volume management. Unfortunately, body fluid composition was not assessed in our study, and further investigations are warranted to clarify this mechanism.

Hyperkalemia is a common and serious concern in MRA therapy. In our cohort, no hyperkalemic crises were observed. Approximately 10% of patients were concurrently prescribed potassium binders such as sodium zirconium cyclosilicate or sodium polystyrene sulfonate to prevent hyperkalemia. In the FIDELIO-DKD trial, which enrolled high-risk patients (mean eGFR 44.3 ml/min/1.73 m^2^; median UACR 852 mg/g), 0.8% of finerenone -treated patients experienced serum potassium ≥6.0 mmol/L, compared to 0.2% in the placebo group [10], with minimal increases in acute kidney injury (AKI; 0.3% vs. 0.2%). In comparison, spironolactone-treated heart failure patients (mean age 73.2 years; mean eGFR 61.8 ml/min/1.73 m^2^; 46.6% with diabetes) experienced hyperkalemia and AKI rates of 2.9 and 10.1 per 1000 person-months, respectively, when co-administered with loop diuretics [30]. In FINEARTS-HF (mean eGFR 61.9 ml/min/1.73 m^2^), the incidence of hyperkalemia (>6.0 mmol/L) and serum creatinine ≥3.0 mg/dL in the finerenone group were 2.0% and 3.0%, respectively, during a median 32-month follow-up [21]. These findings suggest that finerenone may pose a lower risk of hyperkalemia and AKI compared to spironolactone.

Switching from another MRA to finerenone appeared to reveal a potentially distinct renal effect profile. Although our analysis did not reach statistical significance, switching to finerenone was associated with a trend toward renal protection, as reflected by the eGFR slope. The absence of a significant effect may be attributable to the limited duration of observation and the small number of patients with prior MRA use. Finerenone is the only MRA with clearly demonstrated efficacy in DKD through large-scale clinical trials. Importantly, each MRA exhibits distinct pharmacological characteristics [16]. Spironolactone, a steroidal MRA, has a long half-life (>20 h) and multiple active metabolites that also bind androgen and progesterone receptors. In contrast, finerenone is a non-steroidal MRA with a short half-life (2–3 h), no active metabolites, and no affinity for other steroid hormone receptors. Furthermore, in rodents, spironolactone accumulates in the kidney at sixfold higher concentrations than in the heart, while finerenone shows similar distribution in both organs [16]. These properties—short half-life, absence of active metabolites, and balanced tissue distribution—may make finerenone preferable in DKD patients at risk for acute kidney injury. Esaxerenone is another non-steroidal MRA with a receptor-binding profile similar to finerenone. However, it is primarily an antihypertensive agent, reducing systolic blood pressure by approximately 10 mmHg and exhibiting a prolonged half-life (19–25 h) [15]. Although its anti-albuminuric and antihypertensive effects are established, its long-term renoprotective potential remains speculative and likely differs qualitatively from that of finerenone. The difference of outcome after finerenone in blood pressure and BMI with or without prior MRAs should be not the direct influence of MRA; rather, prior MRA use group were much intensively monitored all biological parameters when compared to those of without.

There are several limitations to this study. First, it was a retrospective, single-center analysis with a small sample size. Given that finerenone was often initiated in patients already exhibiting progressive DKD, generalizing these findings to milder cases with normo- or microalbuminuria and preserved renal function may be inappropriate. Our sample size was also insufficient for robust subgroup analysis. Nonetheless, a key strength of our cohort is that most patients were already on SGLT2 inhibitors—unlike the FIGARO-DKD and FIDELIO-DKD trials, where concomitant use was limited. Additionally, the analysis of patients switched from other MRAs to finerenone offers novel insights into finerenone’s distinct renal effects.

## Conclusion

Finerenone may confer significant renoprotective benefits in real-world clinical practice, particularly when added to current DKD regimens that include SGLT2 inhibitors. These findings support the emerging role of finerenone in advancing DKD management in the “post-SGLT2 inhibitor era”.

## Data Availability

The dataset generated and analyzed during the current study are available from the corresponding author on reasonable request.
